# Results of a Qualitative Study to Develop a Patient Reported Outcome Measure for Patients with 4 Subtypes of Soft Tissue Sarcoma

**DOI:** 10.1155/2017/6868030

**Published:** 2017-05-14

**Authors:** Anne M. Skalicky, Sameer R. Ghate, Jose Ricardo Perez, Anne M. Rentz

**Affiliations:** ^1^Evidera, Seattle, WA, USA; ^2^Novartis Oncology, East Hanover, NJ, USA; ^3^Evidera, Bethesda, MD, USA

## Abstract

**Objective:**

The objective of this research was to develop a disease-specific symptom inventory for soft tissue sarcoma.

**Methods:**

Literature review and clinical expert and patient interviews were conducted to determine disease-specific symptoms important to patients with one of the four STS subtypes. Clinical experts identified the most relevant STS symptom items from the item pool developed from literature review. Concept elicitation interviews were conducted with patients to elicit their STS symptom experiences followed by a completion of the draft symptom list via web survey. A cognitive interview was conducted on the comprehension and importance of the symptom items.

**Results:**

Eighty-three symptom items were compiled and discussed with three clinical experts who identified 26 symptoms specific to the four STS subtypes. A total sample of 27 STS participants with self-reported leiomyosarcoma (74%), undifferentiated sarcoma (15%), synovial sarcoma (7%), or liposarcoma (4%) diagnosis completed the web survey and 10 were interviewed. The draft 12-item STS-specific symptom inventory includes abdominal pain, pressure in abdomen, early satiety, bloating, gastrointestinal pain, muscle pain, bone pain, heavy menstrual flow, shortness of breath, chest pain, cough, and painful menstruation.

**Conclusion:**

A number of symptoms are common across STS subtypes and may form a single STS symptom inventory.

## 1. Introduction

Soft tissue sarcomas (STSs) are a heterogeneous group of rare and often malignant tumors that originate from mesenchymal tissue. There are over 50 types of STS identified by the World Health Organization (WHO), and they can arise potentially from every part of the human body. Of all soft tissue sarcomas, approximately 5%–24% are leiomyosarcoma or undifferentiated, 11% are liposarcomas, and 3% are synovial sarcomas [1–3]. More than 50% of STSs develop in an upper or lower extremity usually as a nonpainful mass that has grown over weeks or months and may not appear until the disease is advanced. As the sarcoma grows bigger, it presses on the nearby organs, nerves, muscles, or blood vessels [4]. Because symptoms of STS may not appear until the disease is advanced, only about 50% of STSs are diagnosed before metastasis. Signs and symptoms of the primary or metastasized tumor may include localized pain, cough, and trouble breathing [4].

Treatment options for patients with advanced STS remain limited, with few agents achieving a high objective response rate [5]. Surgery is the main treatment option for most cases of STS, as it is the only potentially curative option [4]. Other treatment options include radiation therapy, chemotherapy, or targeted therapy. For patients with advanced/metastatic disease, the treatment intention is mainly palliative although, with the recent introduction of new approved agents, a second-line therapy is now a reality [6, 7].

To evaluate the benefit of advanced/metastatic STS treatment, it is important to examine whether the treatment is associated with a reduction in symptoms. Thus, guidelines for clinical trials of cancer treatments recommend the inclusion of patient reported outcome (PRO) instruments to support clinical trial endpoints. For the improvement of signs and symptoms assessments to be used as primary endpoints to support cancer drug approval, the FDA should be able to distinguish between improvement in tumor symptoms and lack of drug toxicity [8]. PRO instruments included in trials should be sensitive to changes resulting from treatment in the context of clinical trials and reflect the experiences of people with the condition, in this case, STS. In order for PRO instruments to be used to evaluate treatment benefit to support label claims, evidence of the relevance of symptoms and impacts on function or quality of life concepts to the target sample is required by the United States Food and Drug Association (FDA) [9].

Very few uses of PRO instruments have been reported in oncology drug development programs and have not been emphasized relative to other endpoints related to survival, imaging, and biomarkers. Cancer drugs often carry substantial toxicities that may affect how people feel and function. Including information on patient symptoms and function in clinical trials can provide a comprehensive understanding of patient experiences and what is meaningful to the patient [10].

Treatment options for patients with advanced STS remain limited, with few agents achieving a high objective response rate [5]. This study focused on PRO development for use as an endpoint in the treatment of patients with specific STS subtypes: leiomyosarcoma, synovial sarcoma, liposarcoma, or undifferentiated. Since no disease-specific PRO measures have been developed for patients with these four STS subtypes, this study sought to identify symptoms of these STS subtypes that are most relevant to develop a strategy for evaluating treatment outcomes. The research study sought to evaluate whether it was possible to adapt an existing cancer-specific PRO symptom measure suitable for use with STS patients according to the established guidance on PROs [9, 11, 12]. Adapting the existing PRO tools or item pools is a valid approach to PRO development [13].

## 2. Methods

**Figure 1:**
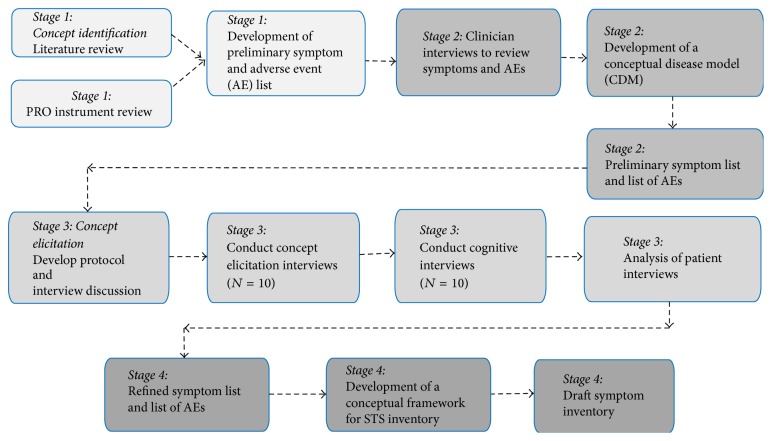
Overview of study stages.

This study involved a multistage PRO development process. An overview of the flow of the project is illustrated in Figure 1. The first stage (concept identification through literature review, PRO instrument content review) and second stage (clinician interviews) resulted in a list of concepts relevant to understand the symptom experience of patients with STS. The information from these two stages was used to develop a conceptual disease model (CDM) of the symptom experience of STS. This CDM was used to identify symptoms specific to the disease and PRO instruments were reviewed for coverage of symptoms to ensure relevance to the patients' experience of STS. These results informed the design of the stage 3 qualitative interviews and web survey with patients with STS to understand their experiences. The patient study was approved by the Chesapeake institutional review board (Columbia, MD) and was conducted in accordance with the Declaration of Helsinki (1964). Finally, in stage 4, the draft PRO instrument was compiled based on findings from stages 1–3 about symptom experience of STS [14].

### 2.1. Stage 1: Identification of STS Symptoms

The stage 1 targeted literature review was conducted to identify relevant randomized clinical trials (RCTs) reporting the use of health-related quality of life (HRQL) or other PROs with STS populations. The search also targeted any previous qualitative studies conducted with STS patients that described symptoms, function, and HRQL experienced by STS patients. Search terms identified relevant published papers through two online services, Medline/PubMed and EMBASE, and all published articles and clinical trial programs were included in the review if they met the inclusion criteria:

- Described results from a clinical trial which included patients with STS;
- Described a qualitative PRO development study including patients with STS;
- Included HRQL, symptom, or function outcome measure;
- Included STS PRO as a primary, secondary, or exploratory outcome measure.

Published articles, conference abstracts, and clinical trial programs were excluded if they were published prior to 2000 or not in English, if they included nonrelevant populations (i.e., children, nonhuman, patient populations not listed above), or if the study was nonempirical or of unspecified methodology (e.g., letters, general review papers/discussion papers, descriptive narratives, and commentaries).

Abstracts of the articles were reviewed for eligibility and subsequently full articles were reviewed to determine final eligibility.

An additional search was conducted to evaluate the content validity of the PROs identified in the literature review. PRO instruments were evaluated against standard criteria for development and validation of PRO instruments [9, 11, 12]. The literature and PRO review findings were used to draft a list of symptoms for review in stage 2 interviews with clinical experts.

### 2.2. Stage 2: Clinicians Identify Important STS Symptoms

Stage 2 of the study focused on obtaining feedback from 3 clinical experts specializing in STS. The clinician interviews were focused on discussion of the interplay of STS subtype, disease progression, stage of cancer and tumor region, and the symptom manifestation of the four STS subtypes. The clinicians were asked to indicate for each symptom whether it was primarily a disease symptom, primarily a treatment symptom, both a disease and a treatment symptom, or not related to disease or treatment.

Prior to the interview, clinical experts completed and returned the symptom questionnaire derived from the stage 1 literature and PRO review. The questionnaire asked the clinicians to indicate whether a symptom was STS-specific only, treatment-specific, related to both STS and treatment, or not related to either STS or treatment. The clinician interviews were conducted by telephone using a semistructured interview guide which included a discussion of the clinical course of the 4 different subtypes of STS as well as clinicians' knowledge of disease-related symptoms affecting these patients. Experts were also asked if symptoms due to an unresectable primary tumor could be distinguished from symptoms due to a metastasized tumor. During the last part of the interview, clinicians' responses to the symptom questionnaire were reviewed.

All interviews were audio-recorded (with permission) and subsequently transcribed. Interviewer notes supplemented the transcripts, capturing key findings from the interviews. The qualitative data obtained during the clinical expert discussion and survey completion were used to develop the conceptual disease model and patient interview discussion guide and refine the STS symptoms list for stage 3 patient interviews. The criterion to include a symptom in the list for stage 3 review by patients was that a symptom must be identified by at least one of the clinical experts as a STS tumor symptom.

### 2.3. Stage 3: Patients Identify Important STS Symptoms

During stage 3, patients were recruited from STS patient advocacy groups or rare patient research panels and invited to complete a web survey and participate in an in-depth telephone interview. The in-depth interview involved two parts: (1) elicitation of STS-related symptoms and (2) cognitive debriefing of the web survey draft symptom items, recall periods, and response options. A target goal was set of 12 interviews with 3-4 patients per STS subtype as well as to have a broad distribution of tumor anatomical locations. Once four interviews were achieved for any of the STS subtype targets, additional participants of that subtype were only eligible to complete the web survey.

To be included in the study, participants had to have diagnosis of advanced primary or metastasized leiomyosarcoma, synovial sarcoma, liposarcoma, or undifferentiated subtypes (histological or cytologic confirmation), previous treatment for STS tumor, and access to a computer with Internet. Exclusion criteria included currently receiving chemotherapy treatment and other major illnesses (serious infection, cardiovascular disease, and liver disease) active in the past 6 months that would limit the ability to assess STS disease symptoms. For telephone interviews, additional inclusion criteria included being able to speak and understand English well enough to participate in an hour-long telephone interview and willing to be audio-recorded during the interview session.

Those patients participating in the in-depth interview were first asked about their STS symptom experience using a semistructured discussion guide. The primary objective of the concept elicitation portion of the interview was to prompt participants to describe their STS-related symptoms. When they mentioned a symptom they experience, they were asked whether they thought the symptom was due exclusively to their STS or tumor (i.e., STS-specific), whether it was because of treatment, or both.

The interviews were audio-recorded and transcribed. The patients' comprehension and understanding of the items, as well as the recall period and response options for the frequency and severity questions, were assessed. After the interview, participants were asked to provide medical release for their clinician to provide diagnosis verification. After the completion of the web survey, those participants scheduled for an in-depth interview participated in a cognitive debriefing interview to assess the selected STS-specific cancer symptom items and the patients' assessment of the frequency and severity of each symptom. A saturation grid captured the symptom concepts experienced in order to identify symptom concepts.

Interested participants completed a web survey which included a list of symptoms developed from stages 1-2. The web survey asked patients to identify and select from a list of symptoms those that were relevant to their STS disease experience. From the selected list, the participants indicated whether the symptoms were STS-specific only (i.e., attributable to tumor), treatment-specific only, both STS and treatment symptoms, or neither STS nor treatment symptoms. Descriptive statistics (mean, standard deviation, and frequency) were used to characterize the symptoms in terms of STS subtype and tumor location.

### 2.4. Stage 4: Finalize STS Symptom Inventory

Based on stages 1–3, a pool of STS-specific symptom items were identified. For an item to be included in the final draft symptom inventory, a priori criteria included symptoms that needed to have been identified as important by at least 50% of patients within a STS subtype or by tumor location in order to capture experiences of half or more of the patients. Additionally, the symptom item had to be amenable to measurement of severity or frequency and possibly sensitive to treatment change within the timeframe of a typical clinical trial according to clinician input.

The draft instrument was developed to encompass both symptom frequency and severity. Instructions, item stems, and response options were derived from patient language elicited during interviews to ensure appropriateness, relevance, understanding, and clarity of the items included in the measure. Response options were reviewed to reflect dimensions that were salient to patient feedback, clinically relevant for the assessment period, and, most importantly, not created de novo but from an existing, established PRO which has undergone patient testing. The recall period needed to be informed by patient interviews as well as the clinical trial setting.

## 3. Results

### 3.1. Stage 1: Identification of STS Symptoms

Overall, 5 of the 57 articles identified as RCTs and 5 of the 283 articles identified as qualitative studies met the inclusion criteria. From these articles, eight existing generic cancer measures that had been used in STS clinical trials were identified (4 HRQL, 4 symptom measures): (1) the European Organization for Research and Treatment of Cancer Quality of Life Questionnaire-Core Questionnaire (EORTC QLQ-C30) [15], (2) MD Anderson Symptom Inventory (MDASI) [16], (3) MDASI-Gastrointestinal Stromal Tumor (GIST) [17], (4) the Standard Form-36 (SF-36) [18], (5) the Toronto Extremity Salvage Score [19], (6) the 3-item Cancer-Related Symptoms Questionnaire [20, 21], (7) the Memorial Symptom Assessment Scale (MSAS), and (8) worst pain numeric rating scale. Using the items from the 8 generic cancer PROs, a list of 44 symptom concepts was compiled.

At the time of this study, the research team became aware of the National Cancer Institute's initiative to develop the Patient Reported Outcomes version of the Common Terminology Criteria for Adverse Events (PRO-CTCAE), a patient reported outcome measurement system of symptomatic adverse events for future use in cancer clinical trials [10]. Thirty-nine adverse event symptoms were identified from the PRO-CTCAE version 1.0 and were included in the symptoms list. The PRO-CTCAE adverse events were cross-checked with the National Comprehensive Cancer Network (NCCN) guidelines for recommended STS treatments and their known adverse events. Overall, the combined symptoms list from the literature review of PROs and list of adverse events from the PRO-CTCAE and NCCN guidelines included 83 items.

### 3.2. Stage 2: Clinicians Identify Important STS Symptoms

Three clinical experts from the US with extensive clinical experience working with leiomyosarcoma, synovial sarcoma, and liposarcoma cancer patients participated in an hour-long telephone interview.

Clinicians primarily described disease symptoms as being more dependent on the tumor stage, location, and size rather than specific histologic subtype. The stage of the tumor, its location, and the degree of nerve, bone, and blood vessel compression drive the symptom experience. In earlier stages of STS, some patients may be asymptomatic and may only present to a physician at a very advanced stage when the tumor is palpable or when the tumor has metastasized to other regions of the body. Initially, we thought the symptom inventory would be specific to the histologic STS subtypes but since the clinicians made it clear that symptoms were more dependent on anatomic location, the data collection instruments were revised to capture more information about the tumor location and a good distribution of patients with tumor from different anatomic locations so as to have a tool with broader relevance across histologic types.

During the open-ended portion of the interview, experts spontaneously named 8 symptoms that were not included in the STS symptoms list developed in stage 1: bone pain, chest pain, early satiety, gastrointestinal pain, heavy menstrual flow, muscle pain, pressure in abdomen, and tumor pain.

During the review of the 83 symptom and adverse event items, the clinicians identified 26 (33%) symptoms as potential STS tumor symptoms (Table 1). Seven (27%) symptoms were selected by all three clinicians: abdominal pain, early satiety, shortness of breath, chest pain, cough, body pain, and edema. Eight (31%) symptoms were endorsed by two clinicians: urinary incontinence, painful menstruation, fatigue, and heavy menstrual flow. Eleven (42%) symptoms were selected by only one clinician: tumor pain, chills, wheezing, heart palpitations, gastrointestinal pain, fecal incontinence, urinary tract pain, decreased libido, depression, difficulty swallowing, and anxiety.

Following the clinician interviews, a 26-item draft STS symptom inventory was developed. The following item criteria were employed: (1) item stem modeled after existing PRO identified in literature review; (2) item reflecting a single concept, rather than multidimensional concept; (3) item wording consistent with patient language; (4) item appropriate for recall period; and (5) response scale corresponding to item concept. The preliminary symptom inventory was modeled after the PRO-CTCAE which has undergone patient testing and includes the measurement of symptom frequency and symptom severity. The symptom frequency stem asked “how often did you feel [symptom]” with response options capturing “never, rarely, occasionally, frequently, and almost constantly.” The symptom severity stem asked “how severe was the [symptom]” with response options “none, mild, moderate, severe, and very severe.”

### 3.3. Stage 3: Patients Identify Important STS Symptoms

During stage 3, a convenience sample of patients were recruited from STS patient advocacy groups (*n* = 21, 78%) or rare patient research panels (*n* = 6, 12%) and invited to participate in the completion of the web survey and an in-depth telephone interview. In total, 17 (63%) STS patients completed the web survey only and another 10 (27%) participated in both the in-depth interviews and the web survey. Of the total sample (*n* = 27), most study participants self-reported leiomyosarcoma (74%) diagnosis, followed by undifferentiated sarcoma (15%), synovial sarcoma (7%), or liposarcoma (4%) diagnosis. Most participants reported primary tumors in the abdominal (31%), pelvic (37%), chest (18%), extremities (7%), and other (11%) regions.

Complete sociodemographic characteristics for the web survey and interview study samples are presented in Table 2. The mean age of web survey sample was 54 ± 8 years and the age of the interviewed study participants was 55 ± 10 years (range: 32–67). The vast majority of the study sample was female and white. Most web survey participants completed a college degree or postgraduate degree (59%) and were disabled (44%) or not working (30%) and 70% reported their current health status as either excellent, very good, or good. The majority of the interview sample completed high school or some college (60%) and were not working (60%) and the majority (80%) reported having excellent, very good, or good health.

#### 3.3.1. Concept Elicitation Interviews

During the concept elicitation interviews, when describing their STS experience, participants mentioned a variety of symptoms. The most common STS tumor symptoms reported were abdominal pain (50%), pressure in the abdomen (40%), cough (40%), chest pain (30%), poor appetite or early satiety (20%), and shortness of breath (20%).  Table 3 includes selected quotes from study participants specific to each STS symptom item.

Out of the ten participants interviewed, seven (70%) reported experiencing abdominal pain with the majority of those (71%) describing their abdominal pain as specifically relating to STS and not due to treatment. When describing their abdominal pain, participants said that it felt like “somebody had punched me in the abdomen” and “it was a bit like labor pain.” One participant stated “I felt nauseous,” and another one described it as “cramping.”

Six of the interview participants (60%) described experiencing early satiety or a sensation of fullness when eating. Only one of the participants with early satiety reported it to be exclusively a tumor symptom. Descriptions of early satiety included that it felt like “bloating” or feeling “just nauseous.”

Half of the participants interviewed experienced pressure in the abdomen. Four of them (80%) said it was a STS symptom. Descriptions of pressure in abdomen included “fullness,” “discomfort,” and “a pushing.” Another participant described pressure in abdomen “as a feeling of your insides pushing outwards.”

Five participants (50%) also experienced gastrointestinal pain. Only two of the five (40%), however, attributed their gastrointestinal pain to STS symptoms: one with synovial sarcoma and the other with undifferentiated sarcoma. Descriptions of gastrointestinal pain included that it felt like “when you're blowing up” and a “stomachache” or “cramp.”

Of the ten participants interviewed, 50% experienced bloating. However, only one (20%) reported it to be exclusively a tumor symptom. Participants described their bloating as “pressure in the abdomen,” “very uncomfortable,” and “pressure when I eat.” A few participants described it as feeling “full before eating anything.”

Six of the ten interview participants (60%) experienced muscle pain but none of them attributed it solely to their STS tumor. When describing their muscle pain, some of the words participants used were “bad pain” and “very painful muscle spasms.”

Sixty percent of interview participants reported bone pain but only two (33%) thought it was exclusively a tumor symptom. Some of the terms used by participants to describe their bone pain included feeling “burning more or less pain” and “just pain.”

Only one of the ten participants interviewed (10%) experienced painful menstruation and she specified it exclusively as a tumor symptom, before her tumor was resected.

Two (20%) of the ten participants interviewed experienced heavy menstrual flow which they both attributed to their STS tumor.

Half of the ten participants interviewed (50%) experienced shortness of breath but only two (40%) reported it to be exclusively a tumor symptom. The participants described shortness of breath as “an asthma attack,” “unable to get air in, unable to get air out,” and “incredible breathing.”

Seventy percent (*n* = 7) experienced chest pain and nearly half of those (43%) reported it to be exclusively a tumor symptom. Descriptions of chest pain included that it felt like “a heart attack,” “like sharp needles,” “discomfort,” “pressure,” and “really bad indigestion.”

Half (*n* = 5) of the ten participants interviewed experienced cough and most (*n* = 4, 80%) reported it to be exclusively a tumor symptom. Descriptions of cough included that it felt like “a tickle in the chest” and “an asthma attack.”

Only 4 (40%) of the 10 participants who were interviewed reported “other symptoms”. They described experiencing STS-related symptoms like bleeding (25%), mouth sores (25%), fluid in their chest (25%), and heartburn (25%). Description of bleeding included that it was bleeding between menstrual periods while mouth sores were described as painful to eat and contributing to a 25 lb weight loss.

The majority of symptom concepts emerged in the first set of interviews for each STS subtype. No new concepts emerged in the last two interviews for the whole sample but we do not assume saturation was reached by either subtype or tumor region because of the small subgroup sample sizes.

#### 3.3.2. Web Survey Sample

**Figure 2:**
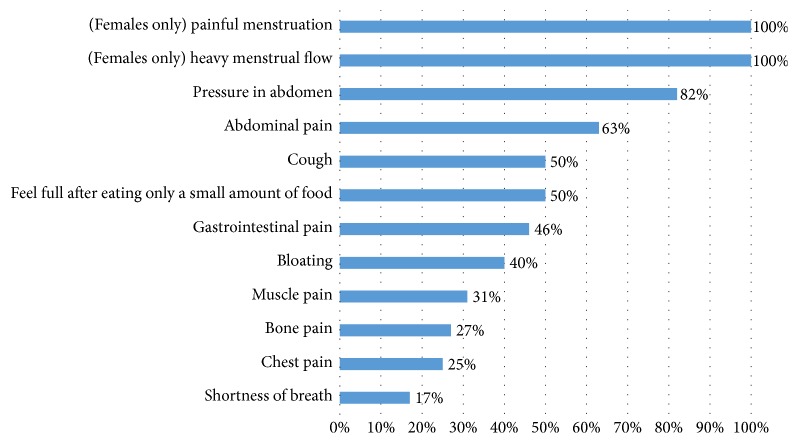
Web survey results for participants reporting symptom as tumor only.

Twenty-seven participants completed the web survey. Web survey participants selected the symptoms they experienced as a result of STS ranging from symptoms like abdominal pain where 70% of the sample experienced this symptom.  Figure 2 includes the breakdown of participants endorsing symptoms and attributing them to STS disease symptoms. The most common symptoms experienced ranged from 70% of the web survey participants reporting abdominal pain to 7% of participants reporting female-only symptoms related to menstruation. Of those experiencing symptoms, the highest percentage attributing the symptom to their STS tumor are reported in Figure 2. The following symptoms are STS tumor symptoms: heavy menstrual flow or painful menstruation (100%), pressure in the abdomen (82%), abdominal pain (63%), early satiety (50%), cough (50%), bloating (40%), gastrointestinal pain (46%), wheezing (33%), muscle pain (31%), bone pain (27%), chest pain (*n* = 3, 30%), and shortness of breath (*n* = 2, 20%). Twelve symptoms were identified by at least 50% of the study participants within a STS subtype group or tumor location group as primarily STS tumor only symptoms.

The results of the importance ranking and mean frequency and severity of each symptom are reported in Table 3. On average, participants ranked chest pain as extremely important (mean ± SD, 3.0 ± 0.3), and gastrointestinal pain (2.8 ± 0.4), muscle pain (2.5 ± 0.6), bloating (2.5 ± 0.5), and painful menstruation (2.5 ± 0.7) were identified as ranging from important to extremely important. Similarly, the symptoms which participants reported as “occasional” during the past 7 days were early satiety, bloating, gastrointestinal pain, and muscle pain. Participants also reported moderate severity for bloating, gastrointestinal pain, and muscle pain.

#### 3.3.3. Cognitive Interview Sample

Table 4 presents the findings from the in-depth interviews with descriptions of the key STS symptoms. In the interviews, participants described frequency of symptoms in terms of “steady,” “continuous,” “frequently,” “fair amount of the time,” “every other day,” or “every day.” The 7-day recall period for the draft frequency items was considered an acceptable timeframe for participants to evaluate change in frequency of many symptoms. Additionally, the frequency response options of “never, rarely, occasionally, frequency, almost constantly” were considered very easy to understand and relevant to study participants.

The interview participants described symptom severity in relation to terms such as “unbearable,” “mild to moderate,” and “severe” symptoms. The severity response options of “none, mild, moderate, severe, and very severe” were considered very relevant and easy to understand to study participants.

### 3.4. Stage 4: Finalize STS Symptom Inventory

The symptom overlap between the literature review and those identified by clinicians and patients were examined. Three symptom items from clinical trials were identified as STS disease symptoms (pain, shortness of breath, and cough) and also were selected by clinicians and patients. Clinicians and patients overlapped on eleven items: early satiety, pressure in abdomen, stomach or intestinal pain, bloating, muscle pain, bone pain, painful menstruation, heavy menstrual flow, and chest pain.

**Figure 3:**
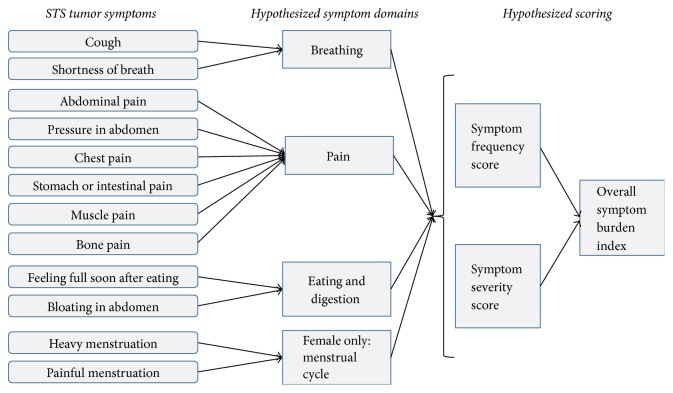
Draft conceptual framework for STS symptom inventory.

The final draft symptom inventory includes 12 symptoms from various anatomical sites, including the extremities: abdominal pain, pressure in abdomen, early satiety, bloating, gastrointestinal pain, muscle pain, bone pain, heavy menstrual flow, shortness of breath, chest pain, cough, and painful menstruation. Two of the twelve items, heavy menstrual flow and painful menstruation, are female-specific. All symptoms include a frequency and severity item. The 12 items are hypothesized to fit into four domains: breathing, pain, eating and digestion, and menstrual cycle. A conceptual framework for the STS symptom inventory is illustrated in Figure 3. And the draft symptom inventory is available in the online supplement (in Supplementary Material available online at https://doi.org/10.1155/2017/6868030). 

## 4. Discussion

The draft STS symptom inventory is designed to evaluate the frequency and severity of STS symptoms in a clinical trial. The challenging aspect of an STS symptom inventory is that it should cover a broad range of symptoms that may affect different regions of the body. Initially, we believed the development process would be driven by the STS histologic subtypes included in the study. But clinicians and patients interviewed spoke to the idea that the symptom experience was driven more by anatomical location of the tumor and less by histological subtype or primary or metastasized status of the tumor.

The stage 1 literature review explored concepts relevant to this patient population resulting in an item bank used to draft a preliminary STS symptoms list for review and discussion with clinical experts. Stage 2 interviews with clinical experts provided in-depth information to develop a conceptual disease model and concept elicitation interview guide for patient interviews, as well as further refinement to the STS symptoms list. Stage 3 consisted of concept elicitation and cognitive interviews with 10 adults and a web survey with 27 adults with self-reported STS to ensure that the symptoms selected in the inventory are relevant and representative for a wide ranging STS patient population. The majority of survey participants and interview participants reported leiomyosarcoma subtype (74% and 40%, resp.), and a third of the participants had tumor locations in the abdomen and pelvic region (30% and 37%, resp.) in the survey. Stage 4 involved an analysis of the qualitative and quantitative data to create the draft inventory. The symptoms of the STS inventory include tumor symptoms experienced in the trunk and extremities.

A 5-point ordinal scale was chosen for the response options for all items because it was the most common response option from the PROs identified in the stage 1 literature review. The response options selected were chosen to reflect dimensions that were salient to all the patients and clinically relevant for weekly assessment. Additionally, and most importantly, the study team selected the frequency and severity response options from the PRO-CTCAE to allow comparison of STS patient symptom reports with adverse events reported due to treatment.

Length of recall period for symptom assessments is frequently a topic of discussion in PRO development [9]. Daily assessment of symptoms is frequently thought to provide the most accurate way to capture participants' experience of symptoms but it is much more burdensome to incorporate a daily measure into a clinical trial. Since patients reported minimal day to day variability of their symptoms, a 7-day recall period was considered acceptable to evaluate change in severity and frequency of many symptoms. The tool is designed to be administered at multiple time points during a clinical trial.

Since the publication of the FDA Guidance for Industry [9], developers of new PROs and those revising existing PROs have benefitted from having a published standard to help guide their way. While we know clinical input is important to ensure that patient tools are reflective of contemporary patient experience and the most current clinical practice, it is also now an expectation that patients will play a very important role in determining the concepts that should be determined for their condition. The instrument development process for the draft STS symptom inventory (including creation of questions, recall period, and response choices) was guided by procedures outlined by the ISPOR PRO Good Practice Task Force, Part II [12], and has been developed thus far in accordance with the FDA guidance [9] including input by patients with STS. And although the study results lead us to believe tumor location is more responsible for a patient's symptom experience than histological subtype, we can only assume the resulting STS symptom inventory is applicable to patients with one of the four specific STS subtypes included in the study. Therefore, while the resulting symptom inventory may not be generalizable to the entire population of patients with STS, we do know it will apply to many because the four subtypes included in the study are the most prevalent.

Although study limitations include reliance on patient self-report for diagnosis and small sample sizes, we have attempted to mitigate these limitations by the examination of multiple sources of data and reliance on existing PRO items and response options. Retrospective confirmation of STS diagnosis was obtained only on half of the study sample by contacting physicians; however, all of the study participants were members of patient advocacy groups or patient panels specific to STS. But reliance on this self-selected convenience sample of STS patients may introduce an unknown bias as to the severity and type of disease symptoms. However, there is some evidence that patient self-report of cancer status and treatment is reliable [22]. Additional clinic-based interviews with synovial sarcoma and liposarcoma patients are an important next step to collect enough evidence of the content validity of the draft STS symptom inventory for patients with clinician-confirmed histologic subtypes. An additional limitation is that a disproportionate number of patients had leiomyosarcoma subtype and 96% of the patients were female. STS is a rare disease and obtaining equal distribution of gender and subtypes of patients was challenging. Further testing will need to be conducted to assess the content validity of the symptom inventory in other subgroups of patients.

## 5. Conclusion

The draft STS instrument was developed to evaluate changes due to treatment in the symptoms of STS in relation to anatomical site of the tumor. The instrument development process (including creation of questions, recall period, and response choices) was guided by published guidelines and procedures [9, 12]. Next research steps include conducting more cognitive interviews with clinician-confirmed liposarcoma and synovial sarcoma patients and patients with tumors in the extremities. Additionally, it will be important to test the psychometric properties of the measure to inform item reduction and the reliability and validity. The intent is to ensure that the final version of the STS inventory is a clinically relevant and psychometrically sound measure of patients' perceptions of tumor symptoms in adults with STS for use as an endpoint in clinical trials and an easy measure for physicians to use to assess treatment impact on symptoms.

## Supplementary Material

The Soft Tissue Sarcoma Symptom Inventory is a 12-item measure of symptom frequency and severity in the past week. The inventory has four hypothesized domains: breathing, pain, eating and digestion and menstrual cycle.

## Figures and Tables

**Table 1:** Results from clinician questionnaires.

Symptom	Clinician-01	Clinician-02	Clinician-03
(1) Abdominal pain	Yes	Yes	Yes
(2) Early satiety	Yes	Yes	Yes
(3) Shortness of breath	Yes	Yes	Yes
(4) Chest pain	Yes	Yes	Yes
(5) Cough	Yes	Yes	Yes
(6) Body pain	Yes	Yes	Yes
(7) Edema limbs	Yes	Yes	Yes
(8) Urinary incontinence	Yes	Yes	No
(9) Constipation	Yes	No	Yes
(10) Painful menstruation	Yes	Yes	No
(11) Fatigue	Yes	Yes	No
(12) Pressure in abdomen	Yes	No	Yes
(13) Heavy menstrual flow	Yes	Yes	No
(14) Muscle pain	Yes	No	Yes
(15) Bloating	Yes	Not sure	Yes

*Note*. Yes: tumor symptom, No: not tumor symptom.

**Table 2:** Self-reported STS participant demographic characteristics.

Participant demographic characteristics	Total study sample (completing web survey) (*n* = 27)	Interview sample (*n* = 10)
Mean age (SD, range)	54 (8, 32–67)	55 (10, 32–67)

Female (%)	26 (96%)	10 (100%)

Race, *n* (%)		
White	25 (93%)	9 (90%)
Other	2 (8%)	1 (10%)

Highest level of education, *n* (%)		
Secondary/high school/some college	9 (41%)	5 (60%)
College degree/postgraduate	18 (59%)	5 (40%)

Employment status, *n* (%)		
Employed, full-time	7 (26%)	1 (10%)
Not working	8 (30%)	6 (60%)
Disabled	12 (44%)	3 (30%)

Current health status, *n* (%)		
Excellent/very good/good	19 (70%)	8 (80%)
Fair/poor	8 (30%)	2 (20%)

STS subtype		
Leiomyosarcoma^1^	20 (74%)	4 (40%)
Liposarcoma^2^	1 (4%)	1 (10%)
Synovial sarcoma^3^	2 (8%)	2 (20%)
Undifferentiated sarcoma^4^	4 (15%)	3 (30%)

Tumor location (primary and/or secondary)^*∗*^		
Abdomen	9 (33%)	3 (30%)
Chest	5 (18%)	3 (30%)
Pelvic region	10 (37%)	2 (20%)
Extremities	2 (7%)	2 (20%)
Other^5^	3 (11%)	2 (20%)

Resected primary tumor		
Yes	24 (89%)	9 (90%)

Metastasized tumor		
Yes	15 (56%)	10 (100%)

^*∗*^More than one location of tumors possible.

^1^Others including retroperitoneal/pubic bone (*n* = 2); and *n* = 1, each of the following tumor locations: interior vena cava; lower back; pelvis/peritoneal tissue; uterus; uterus/abdomen; uterus/abdomen/spine; uterus/fatty layer of colon; uterus/scalp/thyroid/skull base/pancreas; vagina; and vagina/vertebrae.

^2^Leg, lung, and spine (*n* = 1).

^3^Leg/foot (*n* = 1); abdomen, mediastinum (*n* = 1).

^4^Abdomen (*n* = 1); chest, underarms (*n* = 1), tongue, and lymph node (*n* = 1); uterus (*n* = 1).

^5^Leg, lung, and spine (*n* = 1); lymph node (*n* = 1); mediastinum (*n* = 1).

**Table 3:** Web survey results for symptom frequency and severity.

Symptom	Study participants reporting symptom *n* (%)	How important is it that this symptom improve for you?^†^	In the past 7 days, how often did you feel X?^††^	During the past 24 hours, how severe was X at its worst?^*δ*^
Abdominal pain	Average (SD, median)	2.3 (0.9, 2.5)	2.2 (1.2, 2)	1.6 (0.9, 1)
Pressure in abdomen	Average (SD, median)	2.3 (0.7, 2)	2.0 (1.3, 1)	1.8 (1.2, 1)
Feel full after eating only a small amount of food	Average (SD, median)	1.9 (0.7, 2)	2.6 (1.5, 2)	2.3 (1.5, 2)
Bloating	Average (SD, median)	2.5 (0.5, 2.5)	3.2 (1.5, 3.5)	3.0 (2.0, 3)
Gastrointestinal pain	Average (SD, median)	2.8 (0.4, 3)	3.3 (1.5, 4)	3.0 (1.7, 3.5)
Muscle pain	Average (SD, median)	2.5 (0.6, 2.5)	3.2 (1.3, 3)	2.7 (1.0, 2.5)
(Females only) heavy menstrual flow	Average (SD, median)	1.7 (1.5, 2)	2.0 (1.7, 1)	2.0 (1.7, 1)
Bone pain	Average (SD, median)	2.3 (0.6, 2)	2.3 (0.6, 2)	2.3 (0.6, 2)
(Females only) painful menstruation	Average (SD, median)	2.5 (0.7, 2.5)	2.5 (2.1, 2.5)	2.0 (1.4, 2)
Shortness of breath	Average (SD, median)	1.5 (0.7, 1.5)	1.5 (0.7, 1.5)	1.0 (0, 1)
Chest pain	Average (SD, median)	3.0 (0, 3)	1.0 (0, 1)	1.0 (0, 1)
Cough	Average (SD, median)	1.5 (0.7, 1.5)	1.5 (0.7, 1.5)	1.5 (0.7, 1.5)

^†^0: not important, 1: somewhat important, 2: important, and 3: extremely important.

^††^1: never, 2: rarely, 3: occasionally, 4: frequently, and 5: almost constantly.

^*δ*^1: none, 2: mild, 3: moderate, 4: severe, and 5: very severe.

**Table 4:** Patient descriptions of STS disease symptoms.

Symptom	Descriptions from patient interviews
Abdominal pain	It, but it was like dull ache that would then become a very, very strong ache, like the worst, um, oh, it was almost like I felt like somebody had punched me in the abdomen, would be the best way to describe it. [50, female, leiomyosarcoma, abdomen]

Pressure in abdomen	Pressure is just a feeling of your insides pushing outwards, whereas sometimes it then gets worse and it actually hurts. [54, female, undifferentiated sarcoma, uterus]

Feel full after eating only a small amount of food	I felt like the food wouldn't go in, I did not feel full—I felt like I needed to eat more, it just couldn't get in there. [51, female, synovial sarcoma, mediastinum, abdomen and lung]

Bloating	I only ate a third of what I should eat, and then it feels like I should have, like, I've eaten like a full-on. [51, female, synovial sarcoma, mediastinum, abdomen and lung]

Gastrointestinal pain	*When you mention stomachache, can you describe that a little bit more, like where exactly was it located and what did it feel like?* Um, it felt low down in my abdominal area, um, you know, I felt nauseous and, um, I guess I sometimes would kind of cramp up a little bit. [61, female, leiomyosarcoma, abdomen]

Muscle pain	It's not sharp pain, it's just like if you overworked a muscle, that kind of pain. [65, female, leiomyosarcoma, lower back]

(Females only) heavy menstrual flow	It started out as spotting all the time, um, and then it just got heavier until, um, I had to wear a pad all the time, um, when I was bleeding a lot, because the tumor was emerging. [57, female, leiomyosarcoma, vagina]

Bone pain occurs	[Bone pain occurs] frequently for how often I experience it, and I'm going to put moderate for the last 24 hours, because I was feeling it in—my hands were hurting, my shoulders are hurting. [65, female, leiomyosarcoma, lower back]

(Females only) painful menstruation	Is painful menstruation related to your tumor or is it a treatment-related side effect? That was for tumor. How important is that the painful menstruation improve? That was important, but it's not life-threatening. [54, female, undifferentiated sarcoma, uterus]

Shortness of breath	Then one morning, this happened really quickly, I had what I thought was an asthma attack, because I was having such a hard time breathing, it was just incredible breathing. [51, female, synovial sarcoma mediastinum, abdomen and lung]

Chest pain	When the tumor was at its largest it was 21 centimeters, very large and I felt chest pain and pressure. [50, female, leiomyosarcoma, abdomen]

Cough	it comes out of nowhere, um, it's like everything dries up all of a sudden—and it's occasional that it happens. [65, female, leiomyosarcoma, lower back]
