# Supplementary material for: Results of a Qualitative Study to Develop a Patient Reported Outcome Measure for Patients with 4 Subtypes of Soft Tissue Sarcoma
Source: Sarcoma. 2017 May 14;2017:6868030. doi: 10.1155/2017/6868030 (PMC5446879; doi:10.1155/2017/6868030)
Supplement: Supplementary file 1 — The Soft Tissue Sarcoma Symptom Inventory is a 12-item measure of symptom frequency and severity in the past week. The inventory has four hypothesized domains: breathing, pain, eating and digestion and menstrual cycle. [file 6868030.f1.pdf]

SOFT TISSUE SARCOMA (STS) SYMPTOM INVENTORY

Please answer each question thinking about your soft tissue sarcoma symptoms.

1. In the past 7 days how <u>often</u> did you feel pain in your abdomen	☐ Never ***(skip to next symptom row)***<br>☐ Rarely<br>☐ Occasionally<br>☐ Frequently<br>☐ Almost constantly
2. In the past 7 days, how <u>severe</u> was the pain in your abdomen?	☐ No abdominal pain<br>☐ Mild<br>☐ Moderate<br>☐ Severe<br>☐ Very severe
3. In the past 7 days how <u>often</u> did you feel full after you ate a small amount of food?	☐ Never ***(skip to next symptom row)***<br>☐ Rarely<br>☐ Occasionally<br>☐ Frequently<br>☐ Almost constantly
4. In the past 7 days, how <u>severe</u> was the feeling of fullness after you ate a small amount of food?	☐ No feeling of fullness after eating a small amount<br>☐ Mild<br>☐ Moderate<br>☐ Severe<br>☐ Very severe
5. In the past 7 days how <u>often</u> did you feel pressure in your abdomen?	☐ Never ***(skip to next symptom row)***<br>☐ Rarely<br>☐ Occasionally<br>☐ Frequently<br>☐ Almost constantly
6. In the past 7 days, how <u>severe</u> was the pressure in your abdomen?	☐ No abdominal pressure<br>☐ Mild<br>☐ Moderate<br>☐ Severe<br>☐ Very severe
7. In the past 7 days how <u>often</u> did you feel bloated in your abdomen?	☐ Never ***(skip to next symptom row)***<br>☐ Rarely<br>☐ Occasionally<br>☐ Frequently<br>☐ Almost constantly

8. In the past 7 days, how severe was the bloating in your abdomen?	☐ No abdominal bloating<br>☐ Mild<br>☐ Moderate<br>☐ Severe<br>☐ Very severe
9. In the past 7 days how often did you feel pain in your stomach or intestines?	☐ Never ***(skip to next symptom row)***<br>☐ Rarely<br>☐ Occasionally<br>☐ Frequently<br>☐ Almost constantly
10. In the past 7 days, how severe was the pain in your stomach or intestines?	☐ No stomach or intestinal pain<br>☐ Mild<br>☐ Moderate<br>☐ Severe<br>☐ Very severe
11. In the past 7 days how often did you feel chest pain?	☐ Never ***(skip to next symptom row)***<br>☐ Rarely<br>☐ Occasionally<br>☐ Frequently<br>☐ Almost constantly
12. In the past 7 days, how severe was your chest pain?	☐ No chest pain<br>☐ Mild<br>☐ Moderate<br>☐ Severe<br>☐ Very severe
13. In the past 7 days how often did you have a cough?	☐ Never ***(skip to next symptom row)***<br>☐ Rarely<br>☐ Occasionally<br>☐ Frequently<br>☐ Almost constantly
14. In the past 7 days, how severe was your cough?	☐ No cough<br>☐ Mild<br>☐ Moderate<br>☐ Severe<br>☐ Very severe

15. In the past 7 days how <u>often</u> did you feel short of breath?	☐ Never ***(skip to next symptom row)***<br>☐ Rarely<br>☐ Occasionally<br>☐ Frequently<br>☐ Almost constantly
16. In the past 7 days, how <u>severe</u> was your shortness of breath?	☐ No shortness of breath<br>☐ Mild<br>☐ Moderate<br>☐ Severe<br>☐ Very severe
17. In the past 7 days how <u>often</u> did you feel muscle pain?	☐ Never ***(skip to next symptom row)***<br>☐ Rarely<br>☐ Occasionally<br>☐ Frequently<br>☐ Almost constantly
18. In the past 7 days, how <u>severe</u> was your muscle pain?	☐ No muscle pain<br>☐ Mild<br>☐ Moderate<br>☐ Severe<br>☐ Very severe
19. In the past 7 days how <u>often</u> did you feel bone pain?	☐ Never ***(skip to next symptom row)***<br>☐ Rarely<br>☐ Occasionally<br>☐ Frequently<br>☐ Almost constantly
20. In the past 7 days, how <u>severe</u> was your bone pain?	☐ No bone pain<br>☐ Mild<br>☐ Moderate<br>☐ Severe<br>☐ Very severe
21. [female only item]: In the past 7 days how <u>often</u> did you have menstrual flow or bleeding?	☐ Never ***(skip to next symptom row)***<br>☐ Rarely<br>☐ Occasionally<br>☐ Frequently<br>☐ Almost constantly

22. In the past 7 days, how severe was the menstrual flow or bleeding?	☐ No menstrual flow or bleeding<br>☐ Mild<br>☐ Moderate<br>☐ Severe<br>☐ Very severe
23. [female only item]: In the past 7 days how often did you have pain during menstruation?	☐ Never ***(skip to next symptom row)***<br>☐ Rarely<br>☐ Occasionally<br>☐ Frequently<br>☐ Almost constantly
24. In the past 7 days, how severe was pain during menstruation?	☐ No pain during menstruation<br>☐ Mild<br>☐ Moderate<br>☐ Severe<br>☐ Very severe
